# Bioactive Ether Lipids: Primordial Modulators of Cellular Signaling

**DOI:** 10.3390/metabo11010041

**Published:** 2021-01-08

**Authors:** Nikhil Rangholia, Tina M. Leisner, Stephen P. Holly

**Affiliations:** 1 Department of Pharmaceutical Sciences, College of Pharmacy and Health Sciences, Campbell University, Buies Creek, NC 27506, USA; n_rangholia0611@email.campbell.edu; 2 Eshelman School of Pharmacy, Division of Chemical Biology and Medicinal Chemistry, Center for Integrative Chemical Biology and Drug Discovery, University of North Carolina at Chapel Hill, Chapel Hill, NC 27599, USA; tina_leisner@med.unc.edu

**Keywords:** ether lipid, alkylglycerol, signaling, signal transduction, glycerolipid, glycerophospholipid, apoptosis, platelet, cancer

## Abstract

The primacy of lipids as essential components of cellular membranes is conserved across taxonomic domains. In addition to this crucial role as a semi-permeable barrier, lipids are also increasingly recognized as important signaling molecules with diverse functional mechanisms ranging from cell surface receptor binding to the intracellular regulation of enzymatic cascades. In this review, we focus on ether lipids, an ancient family of lipids having ether-linked structures that chemically differ from their more prevalent acyl relatives. In particular, we examine ether lipid biosynthesis in the peroxisome of mammalian cells, the roles of selected glycerolipids and glycerophospholipids in signal transduction in both prokaryotes and eukaryotes, and finally, the potential therapeutic contributions of synthetic ether lipids to the treatment of cancer.

## 1. Introduction

Much like many other types of organic molecules, lipids are essential for life—even ancient life. Bacteria and Archaea kingdoms each synthesize a wide variety of structurally diverse lipids, including many of those conserved in eukaryotes such as phospholipids and sphingolipids [1] All lipids ranging from Archaea to Eukaryota can be divided into eight categories: fatty acyls, glycerolipids, glycerophospholipids, sphingolipids, sterol lipids, prenol lipids, saccharolipids and polyketides [2]. Important categories like glycerophospholipids can be further subdivided into classes and subclasses according to various features including the number of radyl chains, the type of polar headgroup and whether ester or ether bonds are present [3].

Many of these lipids are required for the formation of the physical barrier known as the lipid bilayer that separates primitive and more complex cells from their ever-changing environments. The crucial nature of the maintenance, composition and permeability of cellular membranes is not debatable, but the fact that many resident lipids in the bilayer also serve as substrates for various enzymes that generate potent molecular signals in multiprotein signaling cascades is not immediately apparent. Since the coining of the term “prostaglandin” by the Swedish scientist Ulf von Euler in the 1930s [4], a multitude of bioactive lipid mediators have been discovered that can act as signaling molecules making this aspect of lipid function no longer considered foreign, but increasingly recognized as a significant part of cellular behavior. Some of these signaling lipids such as diacylglycerol (DAG) and lysophosphatidic acid (LPA) are endogenously produced by the action of many isoforms of phospholipase enzymes, while others are derived from the oxidation of essential, polyunsaturated fatty acids (PUFA), including arachidonic acid, ω-3 or ω-6 fatty acids. PUFA can be further oxidized into oxylipins, a large group of signaling lipids that regulate an expansive array of metazoan vascular responses such as platelet aggregation and clot resolution, innate immunity and inflammation [5,6,7]. Bacteria also utilize lipids for signaling purposes, but as with eukaryotes, ether lipids are relatively understudied [8,9,10,11].

In this review, the role of lipids as signaling molecules is analyzed from the lens of one particular class of lipid found in all domains of life from the ancient Archaea to the more complex Eukarya: the understudied and enigmatic ether lipid. Due to the presence of other timely and comprehensive reviews [12,13,14], however, we will focus mainly on the signaling properties of naturally derived glycerolipids and phosphoglycerolipids, their synthetic mimetics designed to interfere with key signaling networks in human disease and briefly, their abundant membrane counterparts, the plasmalogens, about which much has been written already.

### 1.1. Ether Lipid Biosynthesis

**Figure 1:**
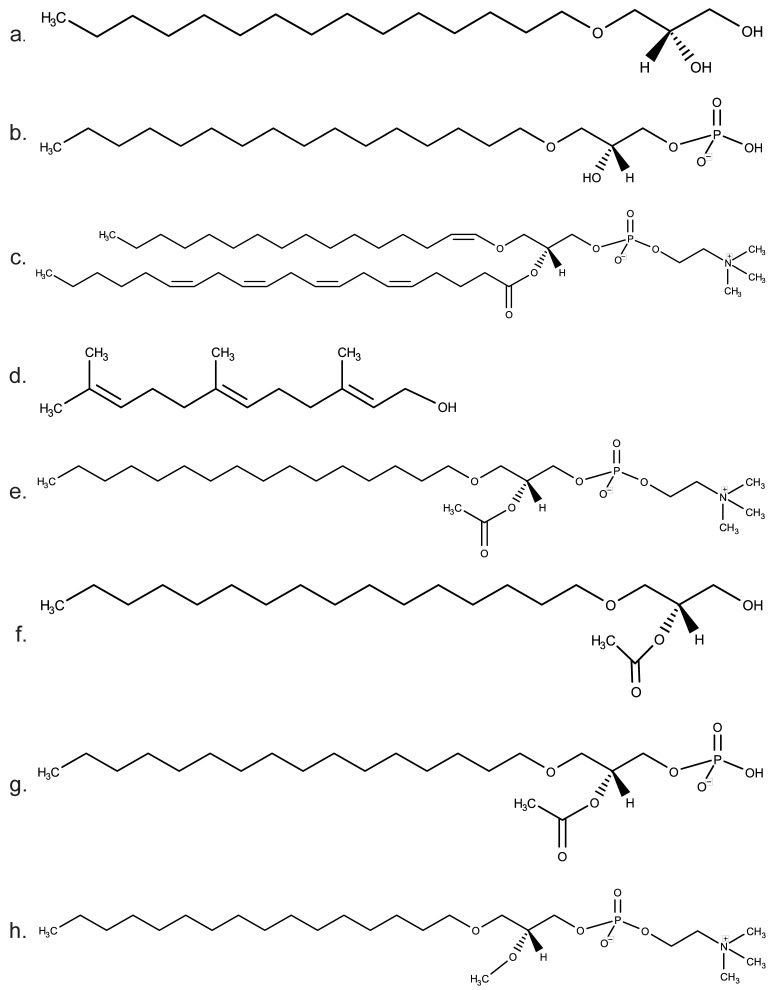
Diverse lipid families contain ether lipids. (**a**). Hexadecylglycerol (HG) is a monoradylglycerol (MG(*O*-16:0/0:0/0:0)) that is a typical alkylglycerol precursor in both prokaryotes and eukaryotes often used to construct more complex ether lipids. (**b**)**.** Alkyl-lysophosphatidic acid or alkyl-LPA (PA(*O*-16:0/0:0) is a group of ether-linked lipids similar to LPA. Both alkyl-LPA and acyl-LPA bind to and activate G protein-coupled receptors, which explains their physiological signaling roles. (**c**). Plasmalogens contain a vinyl ether bond at *sn*-1, usually an unsaturated acyl group at *sn*-2 and either PE or PC at *sn*-3. Shown here is 1-(1*Z*-octadecenyl)-2-arachidonoyl-*sn*-glycero-3-phosphocholine. (**d**). Bacterial triglycerides often contain isoprenoid units of branched unsaturated hydrocarbon chains attached via ether bonds. Shown is free farnesol without ether bonds to glycerol. (**e**)**.** Platelet activating factor (PAF) is a group of choline-containing ether lipids that mainly promote pro-inflammatory signaling pathways. The acetyl group at *sn*-2 required for this activity is added to alkyl-LPC by an acetyltransferase. (**f**)**.** Hexadecylacetylglycerol (HAG) is a diradylglycerol (DG(*O*-16:0/2:0/0:0)) that is the acetylated form of HG (Panel a. above). (**g**)**.** HAG can be phosphorylated to generate 1-*O*-hexadecyl-2-acetyl-*sn*-glycero-3-phosphate (HAGP) shown here, which is thought to compete with DAG to regulate PKC signaling and/or membrane localization. (**h**). Edelfosine is a synthetic anticancer ether lipid that differs from alkyl-PC and PAF in its methyl group at *sn*-2 (1-*O*-octadecyl-2-*O*-methyl-sn-glycero-3-phosphocholine).

Ether lipids or alkyl lipids are any lipids having an ether bond instead of the more prevalent ester bond found in the majority of lipid classes. Alkylglycerols and phosphorylated alkylglycerols are a special type of glycerolipid having an ether bond at the *sn*-1 position of the glycerol backbone, a hydroxyl group or acyl hydrocarbon chain at the *sn*-2 position and a hydroxyl group (alkylglycerol) or a phosphoryl group at the terminal *sn*-3 position (e.g., 1-*O*-alkyl-*sn*-glycerol, Figure 1a or 1-*O*-alkyl-*sn*-glycero-3-phosphate, Figure 1b). To avoid confusion caused by the many different nomenclatures for lipids and their metabolizing enzymes, common lipids or groups of lipids are described using their systematic names, whereas individual lipids with particular chain lengths and double bond configurations will be referred to by their numerical common name or LipidMaps nomenclature. Enzymes are referred to by their traditional substrate/activity names, coupled with their official gene symbol in parentheses when appropriate.

**Figure 2:**
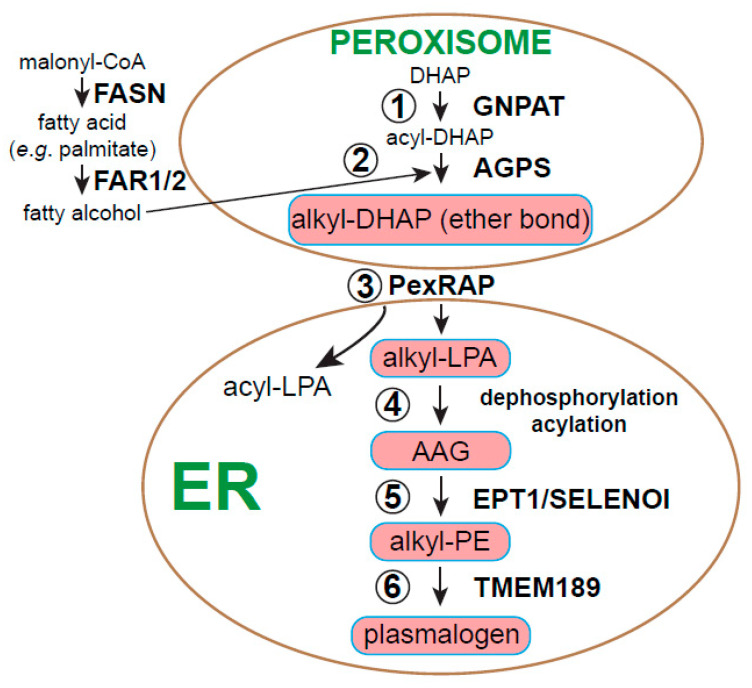
Ether lipid biosynthesis occurs in the peroxisome and ER starting with dihydroxyacetone phosphate (DHAP). (1) DHAP is formed within the lumen of the peroxisome and becomes acylated by glyceronephosphate acyltransferase (GNPAT) to yield acyl-DHAP. (2) Alkylglycerone phosphate synthase (AGPS) replaces this acyl moiety of acyl-DHAP with an alkyl group derived from fatty alcohols, which are products of fatty acyl-CoA reductase 1 and 2 (FAR1/2). This reaction produces the first ether lipid precursor, alkyl-DHAP. (3) A membrane-spanning enzyme, peroxisome reductase activating PPARγ (PexRAP), reduces alkyl-DHAP and acyl-DHAP to alkyl-LPA (Figure 1b) and acyl-LPA respectively, before transporting these products into the ER for further processing. (4) Alkyl-LPA is dephosphorylated and acylated at *sn*-2 in the ER to form alkylacylglycerol or AAG. (5) The ethanolamine phosphotransferase EPT1 adds a phosphatidylethanolamine (PE) group to AAG using CDP-ethanolamine as the polar head group donor, which produces alkyl-PE or plasmanyl-PE. (6) Finally, TMEM189 desaturates the ether bond at *sn*-1 of alkyl-PE to produce the vinyl ether bond characteristic of plasmalogens. PC-containing plasmalogens are formed by exchange of PE for PC by other enzymes (not shown).

In mammals, ether lipids constitute about 20% of the total phospholipid mass [15]. The early steps of ether lipid biosynthesis occur in the peroxisome, a small membrane-bound organelle that transiently interacts with the endoplasmic reticulum (ER) [12,14,16]. The process of ether lipid biosynthesis begins when glycerol 3-phosphate (G3P) is imported into the lumen of the peroxisome and converted to dihydroxyacetone phosphate (DHAP) by G3P dehydrogenase (GPD1). DHAP serves as the substrate for a DHAP acyltransferase known as glyceronephosphate *O*-acyltransferase (GNPAT/DHAPAT/DAPAT) that uses activated fatty acids in the form of acyl-CoA to create an acyl-DHAP product (Figure 2). Acyl-DHAP is the substrate for another peroxisomal enzyme, alkylglycerone phosphate synthase (AGPS), which exchanges the nascent acyl group with a fatty alcohol to form the characteristic ether linkage at the *sn*-1 position as opposed to the more common ester linkage. The fatty alcohol used by AGPS is derived from the reduction of endogenous acyl-CoAs by a membrane-bound protein called fatty acyl-CoA reductase (FAR1/2) or from pools of fatty alcohols obtained from the diet. Interestingly, a pool of fatty acid synthase (FASN) is localized to peroxisomes that produce fatty acids (e.g., palmitate), which are subsequently activated by an acyl-CoA synthetase and reduced by FAR1/2, imported into the peroxisome and incorporated into ether lipids [17]. As discussed later in the oncogenesis section, genetic disruption of either GNPAT or AGPS axes causes a major, albeit not complete, reduction in ether lipid production. These loss-of-function data offer powerful insights into the major contributions of both peroxisomes and the ether lipidome as a whole, but usually do not reveal causal mechanisms centered on individual signaling pathways or lipid species [18,19]. 

The products of AGPS, various alkyl-DHAPs, are simultaneously reduced and exported from the peroxisome en route to the ER as 1-*O*-alkyl-2-lyso-*sn*-glycero-3 phosphate or alkyllysophosphatidic acid (alkyl-LPA). Even though this pathway has been known for quite some time, the reductase/lipid transporter responsible termed peroxisome reductase activating PPARγ (PexRAP or DHRS7B) has only recently been identified in mice [20]. Acyl products of GNPAT that are not substrates for AGPS can also be reduced and exported from the peroxisome as LPA; however, most LPA is produced from lysophosphatidylcholine (LPC) in the blood by autotaxin, a circulating enzyme with phospholipase D-like activity [21,22]. Alkyl-LPA is further modified in the ER, undergoing acylation at the *sn*-2 position by an acyltransferase distinct from GNPAT and dephosphorylation at the *sn*-3 position by a phosphatidate phosphatase activity. This alkylacylglycerol (AAG) intermediate serves as a substrate for the enzyme ethanolamine phosphotransferase 1 (EPT1/SELENOI), which uses CDP-ethanolamine to add an ethanolamine group to AAG to make alkyl-PE, otherwise known as plasmanylethanolamine (plasmanyl-PE) [23]. EPT1 is mutated in certain patients with neurological disorders who display abnormal myelination of neurons and abnormal brain development [23,24]. Skin fibroblasts isolated from these patients showed reduced long-chain acyl-PE and alkyl-PE synthesis. Interestingly, skin fibroblasts synthesized normal levels of the major ester-linked lipids PC and PE, yet accumulated ether-linked PC (plasmanyl-PC), inferring that AAG substrates for EPT1 are repurposed by choline phosphotransferases. Similar reductions in PE were detected in HeLa cells bioengineered to lack EPT1 [23] or in yeast cells expressing human EPT1 with an R122P point mutation, which showed greatly reduced enzymatic activity [24]. Thus, EPT1 is the dominant enzyme responsible for long-chain plasmanyl-PE biosynthesis, which appears to be essential for normal neurological development. 

Alkyl-PE can be subsequently oxidized by plasmanylethanolamine desaturase 1 (PEDS1 or TMEM189) to create a double bond at *sn*-1 (vinyl ether bond) and the final product, 1-alkenyl-PE or plasmenylethanolamine (1-(1*Z*-alkenyl)-2-acylglycerophosphoethanolamine) [25,26]. Similar to EPT1, TMEM189 was only recently identified, which underscores the relative lack of attention and subsequent knowledge of ether lipid metabolism compared to the Kennedy pathway or other well-documented acyl-lipid pathways for phospholipid biosynthesis. The final lipid products of TMEM189 are known as plasmalogens (Figure 1c and Figure 2), which represents the largest group of ether lipids known in eukaryotes, estimated to be over 50% of the total phospholipid content of certain brain regions [15]. We will return to these important ether lipids to briefly evaluate their signaling potential at the end of this review. 

Due to the substrate specificity of TMEM189, however, ether-linked-PC species are thought to be derived from plasmenyl-PE. The phosphoethanolamine group of plasmenyl-PE is removed by phospholipase C to yield 1-alkenyl-2-acyl-*sn*-glycerol, which is a substrate for choline phosphotransferase 1 (CHPT1 or CPT1) or a related enzyme with broader substrate usage such as choline/ethanolamine phosphotransferase 1 (CEPT1). Like EPT1, CEPT1 can also generate alkyl-PE from AAG, but EPT1 seems to be more clinically significant, since no disease-related mutations in CEPT1 have yet been identified [24,27].

### 1.2. Ether Lipid Physical Characteristics

Ether lipid biosynthesis across Archaea and Bacteria domains is distinct. Archaeal membranes contain an abundance of unique ether lipids typified by isoprenoid chains (Figure 1d) linked to glycerol backbones via diether bonds in a 2,3-*sn*-glycerol stereochemistry (isoprenoid dialkylglycerol diether or isoDGD) [28]. Tetraether lipids also exist in Archaea, with two dialkyl glycerols covalently linked by extended isoprenoid units. Tetraethers and other alkylglycerols can be found in Bacteria as well, but these lipids follow a 1,2-*sn*-glycerol stereochemistry similar to eukaryotic glycerolipids [29]. These complex ether linkages are thought to confer reduced ion permeability, increased melting temperature and heat resistance to archaeal and bacterial membranes. Indeed, many thermophilic, halophilic and acidophilic species contain ether lipid-rich membranes presumably as an adaptation mechanism. Since ether lipids can be found in mesophilic and other types of bacteria not living in extreme environments, however, ether lipids may have attained more diverse functions beyond environmental adaptation.

As in prokaryotes, eukaryotic ether lipids are more chemically stable than their acyl counterparts, in large part due to their resistance to lipases, which do not hydrolyze ether bonds but readily cleave ester bonds found in most glycerolipids and phosphoglycerolipids. Even though eukaryotes typically do not inhabit extreme environments, the physico-chemical characteristics imparted by ether bonds may be conserved with respect to altering membrane fluidity and dynamics. For instance, certain ether lipid precursors such as 1-*O*-hexadecyl-*sn*-glycerol (Figure 1a; HG or MG (*O*-16:0/0:0/0:0)) may negatively impact retrograde vesicular transport from the Golgi to the ER, which could potentially reduce intracellular trafficking of endocytosed microbial toxins such as Shiga toxin [30]. This concept of ether lipids altering the physical chemistry of biomembranes may be a central theme for this class of lipids and has been reviewed in depth previously [14]. 

### 1.3. Signaling Ether Lipids in Bacteria

In addition to their apparent function in the lipid bilayer, ether lipids in bacteria were presumed until recently to be static, storage forms of energy-producing fatty acyls [9]. However, bacteria produce a wide variety of ether-linked phospholipids and alkylglycerols that appear to play signaling roles in response to environmental stresses such as starvation. For example, the gram-negative soil-dwelling myxobacteria (*Myxococcus xanthus* and *Stigmatella aurantiaca*) produce fruiting bodies rich in ether lipids that aid in subsequent differentiation into spores. In *M. xanthus*, two distinct metabolic pathways converge on the synthesis of these ether lipids, starting with the formation of the saturated iso-branched fatty acid, 13-methyltetradecanoic acid (FA(i15:0)), which is incorporated into unique branched plasmanyl and plasmenyl phosphatidylethanolamines, including 1-*O*-13-methyl-1-*Z*-tetradecenyl-2-(13-methyltetradecanoyl)-glycero-phosphatidylethanolamine, otherwise known as vinyl ether lipid phosphatidylethanolamine or VEPE [9,11]. According to Ring et al., VEPE is metabolized through several reactions to a monoalkyldiacyl triglyceride called TG1 (TG(*O*-i15/i15/i15) or 1-*O*-(13-methyltetradecyl)-2,3-di-(13-methyltetradecanoyl) glycerol) during nutrient deprivation experiments [11]. TG1 and other alkylglycerols accumulate under these starvation conditions in both *M. xanthus* and *S. aurantiaca*, suggesting a conserved biosynthetic pathway [8,9,10,11]. Indeed, both species of myxobacteria contain a highly homologous cluster of “ether lipid biosynthesis” (*elb*) genes that appear to encode for enzymes critical for synthesizing important ether lipids such as TG1, and other less characterized alkylglycerols. Insertional mutagenesis of the *elbD* gene in both *M. xanthus* and *S. aurantiaca* showed that TG1 formation is completely abolished after 24 h of nutrient deprivation, which correlated with dramatically reduced fruiting body and spore development [10]. The time course of TG1 accumulation and decline was distinct compared to other triglycerides, which is consistent with a role for TG1 as a signaling lipid instead of an energy storage depot [31]. Further evidence for an essential role of TG1 in these developmental programs comes from experiments in which TG1 is exogenously added to bacteria under starvation conditions. TG1 rescued fruiting body formation in *M. xanthus* strains engineered to lack (1) key metabolic pathways for iC15:0 biosynthesis [11], (2) “E signals” involved in inducing sporulation [8] or (3) multiple *elb* enzymes [10]. Even though these data implicate ether lipids like TG1 as essential signaling molecules needed for the execution of complex myxobacterial life cycles, the complete metabolic pathways and molecular targets of these lipids are not yet clear. Discovery of ether lipid targets in this system may be broadly applicable to other cell types such as mammalian adipocytes and may therefore shed light on universal ether lipid signaling mechanisms, especially if those targets are conserved among other prokaryotes and eukaryotes.

### 1.4. Mammalian Ether Lipid Signaling: PAF 

Arguably, the most prototypical ether lipid is platelet activating factor (PAF), a group of 1-*O*-alkyl-2-acetyl-*sn*-glycero-3-phosphocholines of varying hydrocarbon length at *sn*-1 and an invariable acetyl group at *sn*-2 (e.g., PC(*O*-16:0/2:0); Figure 1e). The term PAF was coined by Benveniste and colleagues when they discovered a factor derived from peroxidase-sensitized leukocytes that potently stimulated histamine release and aggregation of purified rabbit platelets [32]. Because the peroxidase-reactive antibody was of the IgE class and histological micrographs revealed massive basophil degranulation and physical association with platelets, the “platelet activating factor” was concluded to have originated from basophils stimulated by IgE-antigen complexes. Around the same time, other investigators were studying potent antihypertensive effects of novel lipids termed antihypertensive polar renomedulary lipid or antihypertensive neutral renomedulary lipid (APRL and ANRL, respectively) [33,34]. When injected intravenously, these lipids caused acute and prolonged reductions in the blood pressure of rats at very low concentrations. APRL later turned out to be identical to PAF [35]. Since those seminal discoveries and the identification of the chemical structure of PAF in 1979, there have been over 14,500 papers published on the structure, signaling properties, pharmacology, physiology and pathophysiology of PAF in animal models and humans. As would be expected for such a vast research field, PAF and the enzymes involved in PAF biosynthesis and catabolism have been the topic of several excellent reviews [36,37,38,39].

#### PAF Biosynthesis and Signaling 

**Figure 3:**
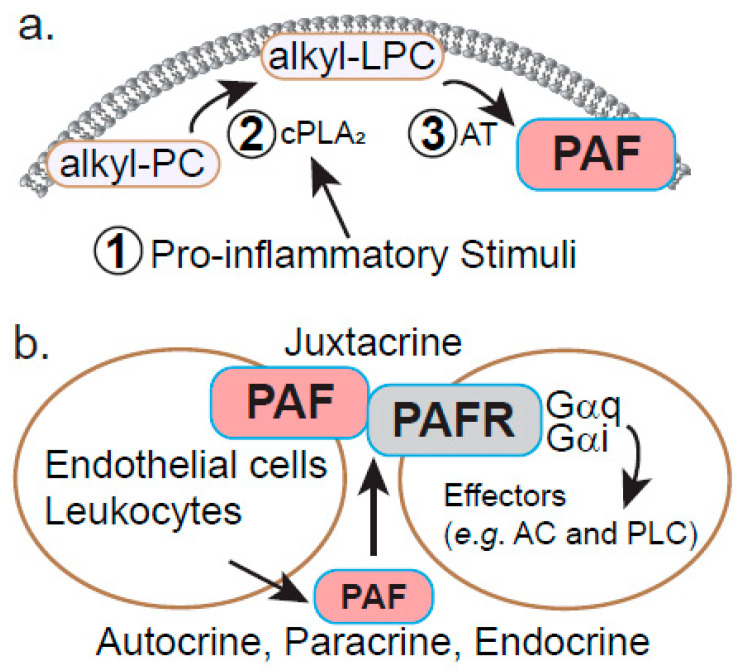
PAF exerts its biological effects via cell-associated/juxtacrine signaling or through secretion. (**a**). Stimulated PAF biosynthesis occurs in cells responding to pro-inflammatory stimuli (1). This causes calcium-dependent activation of cPLA_2_, which cleaves *sn*-2 arachidonyl groups from ether-linked PC species (alkyl-PC) and produces alkyl-lyso-PC (alkyl-LPC), while simultaneously initiating eicosanoid synthesis through arachidonate (2). PAF can also be derived from PC-containing plasmalogens (not shown). (3) Finally, alkyl-LPC serves as a substrate for an acetyltransferase such as LPCAT to generate PAF. (**b**)**.** PAF can signal through several mechanisms: in endothelial cells and certain leukocytes, PAF remains largely cell-associated and interacts with a GPCR (PAFR) on target cells to promote signaling downstream of the Gαq and Gαi heterotrimeric G proteins. Alternatively, PAF can be secreted and interact with PAFR locally (autocrine or paracrine) or distally (endocrine) to stimulate inflammation.

PAF was the first signaling ether lipid discovered and has several modes of action. PAF synthesis begins in the peroxisome (as with all ether lipids) and ends in the ER. PAF is made in a variety of cells, including platelets, myeloid leukocytes such as basophils and monocytes, as well as endothelial cells via two distinct pathways: a two-step remodeling pathway that features cytoplasmic phospholipase A_2_ (cPLA_2_)-mediated cleavage of PC followed by transacetylation by lyso-PAF acetyltransferase (LPCAT1; Figure 3a) [38] and a de novo pathway using alkylglycerol intermediates first described by Snyder and colleagues [40]. The remodeling pathway appears to predominate in leukocytes and endothelial cells since genetic deficiency of cPLA_2_ in mice results in an almost complete loss of PAF biosynthesis in response to the phorbol ester, phorbol myristate acetate (PMA), or the calcium ionophore A23187 [41,42]. Since cPLA_2_ specifically targets arachidonyl-containing PCs as substrates, lipid mediators derived from arachidonate (FA(20:4)) such as eicosinoids (e.g., prostaglandins, leukotrienes, etc.) were also nearly absent in these knockout mice, demonstrating the tight link between PAF and eicosanoid metabolism. In the remodeling pathway, PAF can be synthesized from either arachidonyl-plasmalogens or alkyl-arachidonyl-PC (plasmanylphosphocholine) precursors (Figure 3a). Each of these substrates is first cleaved by cPLA_2_ to generate the lyso species: lysoplasmalogens or lyso-PAF (lyso-alkyl-PC or LPC) respectively. Lysoplasmalogens then undergo a reduction of the vinyl bond at *sn*-1 to form lyso-PAF at which point the two biosynthetic routes converge and lyso-PAF becomes acetylated at *sn*-2 to form the final product PAF. This route is thought to comprise the majority of PAF synthesis in response to inflammatory stimuli and likely accounts for detectable PAF bioactivity in a variety of physiological processes related to acute and chronic inflammation.

Unlike the remodeling pathway, the de novo route of PAF biosynthesis was originally thought to be a constitutively active pathway that established basal levels of PAF in various tissues. PAF de novo synthesis is initiated by the acetylation of alkyl-LPA (Figure 1b) by an acetyl-CoA acetyltransferase to yield 1-alkyl-2-acetyl-*sn*-glycero-3-phosphate. This acetylated species is dephosphorylated by a phosphohydrolase to generate 1-alkyl-2-acetyl-*sn*-glycerol (Figure 1f), which is the substrate for a microsomal choline phosphotransferase [40]. This choline phosphotransferase is dithiothreitol (DTT)-insensitive, which biochemically distinguishes it from previously characterized choline phosphotransferases that are sensitive to DTT and that utilize DAG as a substrate [43]. Even though this DTT-insensitive choline phosphotransferase is considered the main regulatory enzyme of the de novo pathway [40,44], its identity is somewhat unclear, since both CHPT1 and CEPT choline phosphotransferases can synthesize PAF in the presence of 0.1–1 mM DTT from a diradyl glycerol substrate [45]. More recent evidence indicates that inflammation can enhance the de novo pathway and generate several ether lipid intermediates that may have signaling properties independent of PAF [46]. Since key enzymes of the remodeling pathway were also enhanced in parallel, however, the relative significance of the de novo PAF pathway remains obscure.

After synthesis in endothelial cells and leukocytes, PAF is transported to the plasma membrane, where it remains largely cell-associated [47,48]. Once displayed on the surface, it interacts with a G protein-coupled receptor (GPCR) known as the PAF receptor (PAFR or PTAFR) on target cells such as neutrophils or platelets [49,50]. Neutrophils are “tethered” to endothelial cells via P-selectin—P-selectin glycoprotein ligand 1 (PSGL1 or SELPLG) interactions to facilitate PAF binding to PAFR. This novel mode of signaling has been termed juxtacrine signaling, which distinguishes cell-cell contact from other signaling mechanisms characterized by diffusible, secreted factors interacting with receptors on distant (endocrine), proximal (paracrine) or autologous (autocrine) cellular targets. PAF can also be secreted by monocytes and operate locally via autocrine/paracrine signaling or act over longer distances in an endocrine fashion (Figure 3b) [51,52,53]. 

PAFR is coupled to intracellular Gαq and Gαi heterotrimeric G proteins, which send distinct yet synergistic signals into target cells such as leukocytes and platelets [54,55,56,57]. Upon receptor engagement, Gαq exchanges GDP for GTP, dissociates from the heterotrimer and directly activates PLCβ, which hydrolyzes membrane lipids such as phosphatidylinositol bisphosphate (PIP_2_) into the two classical second messenger products: inositol trisphosphate (IP_3_) and the signaling lipid DAG. IP_3_ activates calcium channels within the ER to transiently increase intracellular calcium levels that execute a multitude of functions such as triggering extracellular calcium entry into the cell (store-operated calcium entry or SOCE) [58]. In platelets, calcium activates a variety of enzymes including DAG-sensitive isoforms of protein kinase C (PKC) and CalDAG-GEFI/RasGRP2, a guanyl-nucleotide exchange factor for the small GTPase Rap1 [59,60,61]. Rap1 associates directly with talin, an abundant cytoskeletal protein that changes the conformation of β3 and β1 integrins on the cell surface of platelets to drive aggregation and subsequent downstream signals from ligated integrins [62]. In immune cells, SOCE activates calcium-sensitive lipases such as cPLA_2_, which is critical for eicosanoid formation and the acute inflammatory response that is the hallmark of PAF signaling.

In addition to Gαq, PAFR stimulates GTP loading of Gαi, but unlike the positive signals initiated by Gαq, the predominant signal from Gαi is inhibitory. Gαi binds and inhibits adenylate cyclase, a 12 transmembrane-spanning integral membrane protein that catalyzes the intracellular formation of another second messenger, cyclic AMP (cAMP). Diminished cAMP levels prevent activation of cAMP-dependent signaling molecules such as protein kinase A (PKA) that block initial stimulatory signals coming from Gαq and other sources. Since PKA is reported to initiate anti-inflammatory signals, the PAFR activation of Gαi suppresses these anti-inflammatory signals while simultaneously inducing pro-inflammatory signals through Gαq [36,38].

### 1.5. PAF-Like Signaling Lipids

If lipid signaling function can be inferred from molecular interactions with known second messengers such as calcium or effector proteins such as PKC, then ether lipid components of the PAF de novo biosynthetic pathway are an emerging class of signaling lipids. In addition to PAF, the ether-linked PAF precursors, 1-*O*-hexadecyl-2-acetyl-*sn*-glycerol (HAG; DG(*O*-16:0/2:0/0:0)), the phosphorylated species of HAG (HAGP; 1-*O*-hexadecyl-2-acetyl-*sn*-glycero-3-phosphate; PA(*O*-16:0/2:0)) and alkyl-LPA have all been shown to possess either direct or indirect signaling properties in various cell types. Some of the more recent examples are detailed below. 

### 1.6. HAG and HG

HAG has been reported to affect the biological activity of many cell types, including porcine endothelial cells, human leukemia cells, smooth muscle cells and platelets [63,64,65,66]. Exogenous HAG monomers inhibit the ability of platelets to associate with each other in a process known as aggregation [63], an essential part of blood clot formation in vivo. HAG also blocks the platelet secretion of intracellular granules that contain secondary agonists such as ADP, which reinforce aggregation via a positive-feedback mechanism [67]. In platelets and ovarian carcinoma cells, HAG is metabolized to HG by an enzyme known as arylacetamide deacetylase-like 1 (AADACL1/NCEH1/KIAA1363). AADACL1 activity is upregulated in invasive breast and ovarian cancer cells and is positively associated with cell growth [68,69]. In addition, AADACL1 expression levels may be indicative of gastric and pancreatic cancers [70,71]. Unlike the addition of HAG or inhibition of AADACL1 activity with the small molecule JW480 in platelets [72], HG does not appear to affect platelet aggregation or secretion. These data suggest that HAG but not HG is an endogenous inhibitory lipid that regulates platelet aggregation and vesicle secretion and that AADACL1 potentially regulates these important cellular events by metabolizing HAG to HG.

The mechanism of action for HAG has been closely linked to PKC by many independent groups. In fact, HAG may interact directly with zinc finger domains called C1 domains within certain PKC isoforms. C1 domains bind phorbol esters and DAG, which is instrumental for PKC localization to cellular membranes and the multistep activation of PKC kinase activity [67,73,74]. HAG and related alkylglycerol binding to C1 domains typically inhibits PKC’s ability to phosphorylate substrates in response to well-characterized activators, such as phorbol esters, DAG or DAG mimetics like 1-oleoyl-2-acetyl-*sn*-glycerol (OAG) [75,76,77,78,79]. The ether bond at the *sn*-1 position of HAG is thought to be important for this inhibition [80], which presumably occurs through a competition with PKC activators. Since C1 domain ligands are thought to disrupt critical autoinhibitory interactions that keep PKC in an inactive confirmation, however, it is unclear how HAG or any other C1 domain ligand could compete for binding to PKC without relieving autoinhibition and promoting kinase activity. Nevertheless, HAG by itself, in the absence of PKC activators, does not stimulate PKC activity and is therefore not a DAG mimetic. Perhaps HAG interacts nonproductively with PKC C1 domains and prevents/reduces DAG binding to maintain PKC isoforms in a catalytically incompetent state. Another possibility is that ether lipids such as HAG block PKC kinase activity by preventing PKC translocation to cellular membranes [81,82]. For example, macrophages pretreated with HAG showed reduced GFP-PKCε localization to phagosomes in response to an Fcγ ligand [81]. Importantly, engagement of Fcγ produces DAG, which is known to be required for PKCε translocation, but this important step in the PKC activation cycle seems to be antagonized by HAG. Likewise, various species of alkylacylglycerols (AAG) purified from shark liver oil antagonize phorbol ester- and calcium ionophore-mediated permeability of albumin across the plasma membranes of cultured endothelial cells, which implies a competition with DAG at C1 domains on PKC [65].

The mechanism of HAG-mediated inhibition may be more complex than a straightforward competition with PKC activators, however, since HAG may also activate PKC, at least in the presence of other molecules such as DAG or phorbol esters [74,83]. Slater et al. showed that treatment of purified PKCα or PKCβI potentiates phorbol ester binding to a distinct site, resulting in lower calcium requirements and slightly enhanced kinase activity. Unlike DAG and OAG, however, HAG does not activate PKC directly in the absence of other molecules. Consistent with this, HAG prepared in vitro from phospholipase C-mediated cleavage of PAF stimulated a PKC-like activity in neuroblastoma cell homogenates in the presence of phosphatidylserine (another stimulatory lipid for PKC). It is not clear, however, which PKC isoforms were present in these samples, or perhaps more importantly, whether other kinases capable of phosphorylating the histone III substrate in the assay were present [84]. Surprisingly, this same group also found that pretreatment with HAG, DAG or OAG blocked phorbol ester binding to smooth muscle cells, suggesting that HAG effects may depend on additional, unidentified cellular factors [84].

In addition to its inhibitory potential, HAG may also have weak agonist properties in human platelets. Platelets pretreated with high concentrations of HAG exhibit modestly enhanced aggregation and calcium flux in the presence of agonists, whereas HAG by itself causes cytoskeletal rearrangement (shape change) in the absence of agonists [67]. One possible mechanism is that HAG is acting as an intermediate in the de novo PAF biosynthesis pathway, leading to relatively low levels of PAF that do not strongly stimulate platelet activation. 

### 1.7. HAGP

**Figure 4:**
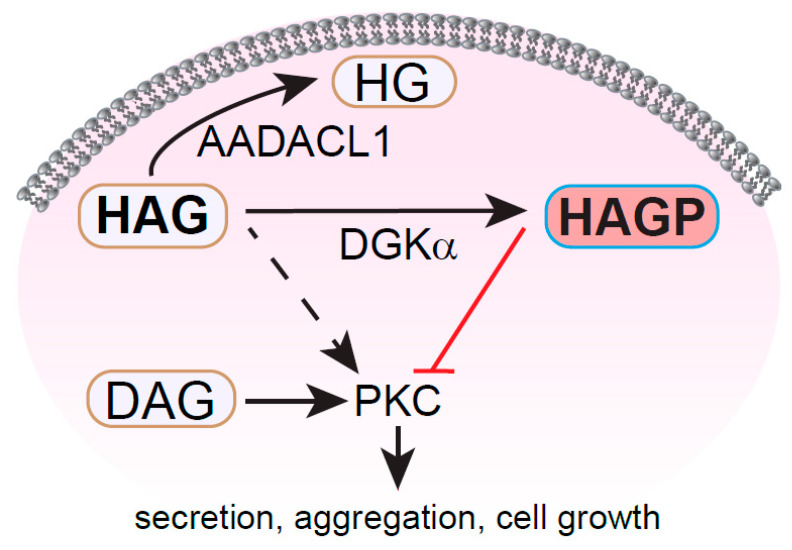
HAG modulates PKC signaling in distinct cell types. In platelets and certain cancer cells, exogenously added HAG is deacetylated by the serine hydrolase AADACL1 to yield its inactive metabolite HG, which may serve as a precursor for other ether lipids and/or contribute to membrane dynamics. In platelets and ovarian carcinoma cells, HAG can also be phosphorylated by a DGKα-like lipid kinase to generate HAGP, which inhibits PKC activity and platelet activation (red line), likely by interfering with DAG binding to PKC isoforms. In contrast, HAG may enhance PKC-dependent cell growth in smooth muscle cells through an unknown mechanism (dashed line). Adapted from Holly et al. [67]

Perhaps much of the seemingly discordant data surrounding HAG can be explained by a single HAG metabolite, HAGP (Figure 1g). In cancer cells, platelets and smooth muscle cells, HAG can be deacetylated to HG by AADACL1 [63,68,84]. In the absence of AADACL1 activity, however, HAG can be rapidly converted to its phosphorylated form, HAGP, by a DAG kinase-like enzyme (e.g., DGKα), in both human platelets and ovarian carcinoma cells [67,85]. HAGP was originally discovered in rabbit platelets by Snyder and colleagues as a major metabolite of HAG that appears within minutes of treatment with radiolabeled HAG [40]. More recently however, the kinetics of HAG to HAGP conversion has been correlated with inhibition of aggregation, secretion and calcium flux in human platelets [67]. Inhibition of DGKα in these cells reversed the inhibitory effects of HAG on platelet aggregation, indicating that the conversion of HAG to HAGP was critical for HAG-mediated inhibition. When incorporated into lipid vesicles, HAGP directly bound to C1a domains from PKCα and PKCδ, and both HAGP monomers and HAGP-containing vesicles decreased phorbol ester-stimulated PKCα activity in vitro [67]. Interestingly, HAG does not appear to be phosphorylated in smooth muscle cells, which results in PAF-like stimulation of PKC and cell proliferation rather than PKC inhibition [84]. Although additional molecular mechanisms may exist, these intriguing data suggest that the expression level and/or activity of lipid kinases balance cellular pools of HAG and HAGP, which in turn directly modulate PKC activity and downstream cellular events such as aggregation/secretion in platelets and cell growth in smooth muscle cells, with increased HAGP/HAG ratios favoring reduced PKC activity. Therefore, HAGP and HAG likely represent novel regulatory nodes targeting PKC and perhaps other signaling molecules during critical cellular events (Figure 4). 

### 1.8. Ether Lipids and Cancer

Other PAF-like ether lipids may control important growth circuits during tumorigenesis and cancer pathophysiology, since ether lipids as a class are upregulated in several distinct types of cancer including brain, breast, ovarian and skin cancers [18,69,86,87]. Natural ether lipids may act as oncogenic ligands for GPCRs, while synthetic ether lipids may have multiple mechanisms related to apoptosis.

#### 1.8.1. Alkyl-LPA

Alkyl-LPA is a family of ether-linked phospholipids with radyl chains of varying lengths and degrees of saturation that mainly differ from their more common acyl-LPA counterparts by their alkyl linkage at *sn*-1 of the glycerol backbone. As mentioned earlier, alkyl-LPA is an intermediate in the synthesis of ether lipids derived from the peroxisome, but it is also a potent agonist at GPCRs. Much like PAF, for instance, alkyl-LPA can potently drive platelet aggregation through ligation of endothelial differentiation gene (EDG) receptors including LPA5 [88,89]. Since alkyl-LPA is present in the lipid core region of atherosclerotic plaques, which upon rupture, can activate circulating platelets, it may play a pivotal role in thrombosis [90,91]. 

In addition to platelet activation, alkyl-LPA has also been implicated in various aspects of tumor progression related to cell movement [18,92]. To study the effect of ether-lipid depletion in carcinogenesis, Benjamin et al. dramatically lowered the expression levels of AGPS, the key enzyme that introduces the characteristic ether bond in the peroxisome that is overexpressed in breast cancer, melanoma, primary human tumors and Ras-transformed cells. Upon short hairpin RNA (shRNA)-mediated knockdown of AGPS in breast 231MFP and C8161 melanoma cells, the authors showed that both alkyl-LPA and its phospholipid metabolites (plasmanyl and plasmenyl species of PE and PC) were reduced in these cancer cell lines [18]. Unexpectedly, lack of AGPS activity decreased levels of non-ether lipids, such as arachidonate, FA(20:4), and palmitate, FA(16:0), as well as eicosinoids derived from arachidonate, such as prostaglandin E_2_ (PGE_2_). As a result of this decrease in both ether and acyl lipid pools, 231MFP cells were less invasive, less migratory and did not produce tumors when transplanted into living mice in xenograft assays. Addback of alkyl-LPA or PGE_2_ rescued these migration and invasion defects, whereas the addition of PAF or other alkyl-phospholipids did not. Conversely, the overexpression of AGPS converted benign cancer cells into more aggressive subtypes with respect to cell migration and serum-free survival [18]. These data link both alkyl-LPA and PGE_2_ to an aggressive, oncogenic phenotype, which is consistent with AGPS controlling an essential lipidomic node within neoplastic signaling networks.

#### 1.8.2. Synthetic Anticancer Ether Lipids

Synthetic, man-made ether lipids inspired by the structure of lysophosphatidylcholine (LPC) have proven to be potent regulators of oncogenic cell growth [93,94]. In 1993, the synthetic ether lipid known as edelfosine (1-*O*-hexadecyl-2-*O*-methyl-*sn*-glycero-3-phosphocholine or ET-*O*-18) was shown by two groups to promote programmed cell death or apoptosis in leukemia cells [95,96]. Eldefosine later proved to be selective for cancerous cells compared to normal cells and thus became the prototype for the development of structurally similar drugs with ether-linked glycerol backbones designed to fight cancer progression, two of which are described below.

#### 1.8.3. Edelfosine

Despite its structural similarity to PAF (methyl vs. acetyl at *sn*-2; Figure 1h), edelfosine does not appear to act as a ligand for cell surface receptors that regulate calcium flux [97]. Consequently, many signaling pathways that contribute to cancer cell death such as apoptosis have been proposed as intracellular targets of edelfosine. Gajate et al. first showed that edelfosine operates through pro-apoptotic mechanisms in HL-60 cells by demonstrating that edelfosine treatment disrupted mitochondrial membrane potential (ΔΨ_m_) and subsequent activation of caspase-3, which are components of the “intrinsic” apoptotic pathway [98]. These authors also observed cleavage of poly(ADP-ribose) polymerase (PARP), DNA fragmentation and cleavage of caspase-3 itself. Moreover, cell-permeable caspase inhibitors efficiently blocked edelfosine-mediated cell death, but did not disrupt ΔΨ_m_ in these cells, suggesting that edelfosine’s ability to alter mitochondrial membrane integrity lies upstream of caspase activation and intrinsic apoptosis. Similar pro-apoptotic results were reported for edelfosine and related ether lipids in multiple leukemic cell lines [99,100]. 

Edelfosine has been implicated in “extrinsic apoptosis” as well, which is distinguished from its mitochondria-centric intrinsic counterpart by the nature of the apoptotic stimuli. Extracellular ligands such as Fas ligand (FASLG) or tumor necrosis factor-related apoptosis-inducing ligand (TRAIL) interact with “death receptors” such as the Fas receptor/CD95 (FAS) or TRAIL receptors respectively, to trigger extrinsic apoptosis. Edelfosine has been proposed to cluster FAS into detergent-resistant lipid rafts independently of the natural ligand FASLG to promote extrinsic apoptosis and cancer cell death [101,102,103]. This mechanism is somewhat controversial, however, since other groups have demonstrated FAS-independent yet edelfosine-dependent modes of cell death [104,105]. 

In addition to apoptotic pathways, edelfosine may target ion channels such as the calcium-gated potassium channel SK3/K(Ca)2.3, which has been implicated in cancer cell migration [106]. Acute treatment of the highly metastatic MDA-MB-435s cell line with 10 µM eldefosine reduced the calcium sensitivity of SK3/K(Ca)2.3 and subsequent cell migration without inhibiting the binding of apamin, a honeybee peptide known to block SK channels, indicating an allosteric mechanism of inhibition. The polar choline group of edelfosine is important for this activity, since neither 1-*O*-hexadecyl-2-*O*-methyl-*sn*-glycerol (HMG) nor HG had any effect on cell migration in HEK cells overexpressing SK3/K(Ca)2.3 [107].

Finally, edelfosine may disrupt signaling pathways that emanate from the most abundant phospholipid in mammalian membranes, phosphatidylcholine (PC), or an equally important group of signaling lipids, the phosphatidylinositols (PI) [108,109]. Edelfosine and other synthetic ether lipids incorporate into biological membranes and inhibit PC biosynthesis in the ER, which can trigger apoptosis [109,110]. Blockade of PC and/or PI synthesis may not only predispose cells to apoptosis, but may also decrease levels of pivotal signaling lipids, such as DAG and second messengers derived from lipase-dependent hydrolysis of these phospholipids. DAG activates “conventional” and “novel” classes of PKC isoforms, which are thought to have opposing influences on apoptosis. In general, novel PKCs (nPKC) induce apoptosis, whereas conventional PKCs (cPKC) protect against apoptotic stimuli [111,112]. For example, PKCδ promotes the secretion of autocrine factors from prostate cancer cells that bind tumor necrosis factor (TNFα) and TRAIL receptors to stimulate extrinsic apoptosis [111]. Conversely, the stimulation of PKCα, a cPKC isoform, prevents apoptosis in bladder cancer and models of leukemia, whereas the inhibition of cPKC isoforms triggers apoptosis [113,114,115,116].

**Figure 5:**
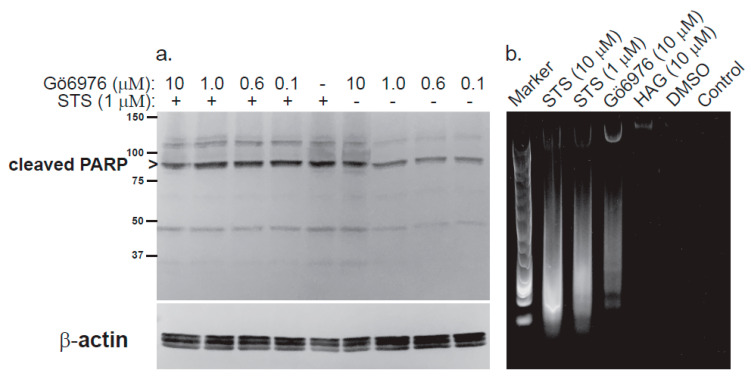
Inhibition of conventional PKC isoforms does not reverse staurosporine-induced apoptosis and causes nucleosome formation in megakaryoblastic cells. CMK11-5 cells were grown in RPMI 1640 media (GIBCO) with 9% FBS at 37 °C in 5% CO_2_. Cells were treated with the indicated concentrations of staurosporine (STS) and/or Gö6976 for 24–48 h, lysed with RIPA buffer (25 mM Tris-HCl pH 7.6, 150 mM NaCl, 1% NP-40, 1% sodium deoxycholate and 0.1% SDS) and assayed for total protein concentration (BCA assay, Thermo). (**a**). Lysates were subjected to SDS-PAGE for Western blotting with a rabbit anti-cleaved PARP polyclonal antibody (#9541, Cell Signaling Technology) to measure apoptosis or a mouse anti-β-actin monoclonal antibody (#3700, Cell Signaling Technology) to measure protein loading. Primary antibodies were recognized by anti-rabbit or anti-mouse IgG-alkaline phosphatase (AP) secondary antibodies for PARP and actin visualization respectively with chromogenic AP substrates. Cleaved PARP runs at approximately 89 kDa, whereas β-actin runs at 45 kDa (*n* = 3). (**b**). Genomic DNA was extracted from the same lysates used in panel a. and separated by electrophoresis on a 0.8% agarose gel. DNA was stained with Diamond Nucleic Acid Dye (Promega) according to the manufacturer’s instructions. Note the DNA ladders present in STS- and Gö6976-treated samples (*n* = 2).

Evidence from our lab supports this antagonistic paradigm between cPKC and nPKC isoforms in cancer cells. In the leukemic megakaryoblastic cell line, CMK11–5 [117,118], the bacterial metabolite and nonselective PKC inhibitor staurosporine caused caspase-dependent intrinsic apoptosis, as measured by proteolytic cleavage of the caspase-3 substrate PARP and genomic DNA fragmentation (Figure 5). Apoptosis was not reversed by the small molecule inhibitor Gö6976, which selectively targets cPKC isoforms PKCα, PKCβ and PKCβII, nor was apoptosis blocked by the previously mentioned ether lipid HAG at 1–20 µM. HAG was rather inert in this system, since it neither caused nor protected against staurosporine-induced apoptosis. Interestingly, Gö6976 alone at the highest concentration tested (10 µM) induced DNA fragmentation/ladder formation comparable to that of staurosporine (Figure 5). These data suggest that conventional PKC isoforms tonically protect against caspase-dependent apoptosis in megakaryocytic cells, but since only small molecule inhibitors were used, this conclusion should be corroborated using more selective methods (e.g., genetics). 

#### 1.8.4. Perifosine

Despite its anticancer efficacy, edelfosine caused gastrointestinal and hemolytic toxicity in clinical trials, which spurred the development of safer and more effective synthetic ether lipids [94]. One such ether lipid currently being evaluated in clinical trials targeting various solid tumors, leukemias and lymphomas is perifosine. The structure of perifosine varies quite significantly from edelfosine in that it lacks the entire three-carbon glycerol backbone and has a dimethyl piperidine group in place of the phosphocholine group of edelfosine. As might be expected from such a deviation from the original edelfosine template, the mechanism of action for perifosine is distinct from edelfosine and involves perturbation of the subcellular localization of the serine/threonine protein kinase, protein kinase B (PKB/AKT). Due to the ability of AKT to antagonize apoptosis and thus promote cell survival in cancer cells, it has been the focus of an intense anticancer strategy for many years [119]. AKT normally localizes to membranes via its pleckstrin homology (PH) domain, which binds specific phosphatidylinositol products such as PI(3,4)P_2_ and PI(3,4,5)P_3_, which are downstream products of the lipid kinase phosphatidylinositol 3-kinase (PI3K). Membrane association facilitates activation of AKT by *trans* phosphorylation on residue T308 by phosphoinositide-dependent kinase (PDK1/PDPK1) and on residue S473 by multiple kinases, including PDK2 and the mechanistic target of rapamycin complex (mTORC). Once activated, AKT dissociates from the membrane and phosphorylates numerous downstream effector proteins, such as the cell cycle protein p21 WAF1/CIP1 (CDKN1A) and the mitochondrial proteins BCL2-associated agonist of cell death (BAD) and X-linked inhibitor of apoptosis (XIAP), which antagonizes apoptosis and promotes cell survival [120,121]. Perifosine treatment of prostate cancer cells results in subcellular mislocalization of AKT, which prevents AKT phosphorylation and subsequent phosphorylation of downstream substrates, all of which leads to cell growth arrest [120]. Expression of a myristoylated (FA(14:0)) AKT variant rescues AKT localization and activity, effectively bypassing perifosine-dependent inhibition of the cell cycle and growth arrest. Due to its ability to incorporate into membranes [109], perifosine presumably competes with PIP_2_ and PIP_3_ at the AKT PH domain and prevents proper AKT localization, resulting in reduced cell survival and increased cell death. Perifosine is currently being tested alone and in combination with various anticancer drugs in Phase I and II trials targeting leukemia, myeloma and other solid cancers [122].

### 1.9. Ether Lipid Metabolism and Other Lipid Pathways

There are many examples of ether lipid biosynthesis pathways that impact seemingly unrelated lipid classes. For example, the alkylglycerol products of AADACL1 (e.g., HG) lead to the production of alkyl-LPA in cancer cells. Similar to disruption of the AGPS axis, reduced protein expression and enzymatic activity of AADACL1 via shRNA-mediated RNA interference lowered HG and downstream alkyl-LPA levels in ovarian carcinoma cells, which correlated with diminished tumor volume in AADACL1-deficient tumors transplanted into nude mice [68]. This metabolic link may not be preserved in non-cancerous cells, however, since murine bone marrow-derived macrophages (BMDM) pretreated with the pharmacological inhibitor of AADACL1, JW480, show increased and not decreased levels of alkyl-LPA [123]. JW480 decreases inflammatory cytokine production (e.g., TNFα) at least partially through alkyl-LPA, which may be acting as an LPA receptor agonist. Interestingly, JW480 treatment also upregulated sphingosine, plasmanyl-PC and plasmenyl-PC, suggesting that AADACL1-dependent ether lipid metabolism has a broad impact on the BMDM lipidome. Whether AADACL1 metabolizes unknown substrates besides HAG or whether HG synthesis impacts multiple lipid classes is not yet clear nor are the mechanisms linking ether-linked phospholipids to cytokine production in pro-inflammatory cells.

Another example of crosstalk between lipid classes comes from cells with reduced alkylglycerol monooxygenase (AGMO) activity. AGMO is expressed in monocytes and macrophages and is the only enzyme capable of breaking the ether bond in alkylglycerols [124]. The reduction of AGMO protein levels and activity in a murine macrophage cell line via RNA interference resulted in predictable increases in alkylglycerols known to be substrates of AGMO such as HG [125]. Surprisingly though, levels of unrelated lipids belonging to the glycosylated ceramide and cardiolipin classes were also found to be elevated, as were many more lipids that could not be precisely identified. Interestingly, prominent ether lipids thought to be AGMO substrates including PAF and lyso-PAF were not altered by AGMO knockdown. Collectively, these data suggest that lowering the activity of an enzyme specifically involved in ether lipid metabolism can have global effects on multiple lipid classes and reveal hidden interrelationships among various biosynthetic pathways of the cellular lipidome. 

### 1.10. Plasmalogens

As mentioned earlier, plasmalogens are the most abundant class of ether lipids and perhaps the most clinically relevant. They have been implicated in a variety of neurological disorders including Alzheimer’s disease, autism, epilepsy, multiple sclerosis, Parkinson’s disease, psychiatric depression, schizophrenia and even ischemic stroke [15,126]. All of these disorders, with the exception of epilepsy, involve reduced levels of plasmalogens in patients, which implies a loss-of-function phenotype and a potentially protective role for these phospholipids under normal physiological conditions. Mechanisms for plasmalogens in the nervous system have been mostly gleaned from knockout mice that do not express proteins required for transport into the peroxisome (e.g., PEX7) or the critical enzymes required for ether lipid biosynthesis, AGPS or GNPAT. These mechanisms range from proper development of essential brain regions such as the cerebellum to myelin sheath formation in neurons [19,127,128,129]. Whether these phenotypes result specifically from plasmalogen deficiency or should be attributed to other types of ether lipids or to a single species of ether lipid is an open question. 

Plasmalogen contributions to signal transduction are even less clear than their influence on neuronal physiology, but may stem from the composition of their fatty acyl chains [13]. Both choline- and ethanolamine-containing plasmalogens are well known to be enriched in acyl-linked PUFA at their *sn*-2 positions, which provide an abundant source of fatty acid precursors upon liberation by cPLA_2_. Free PUFA are oxidized by several enzyme families, such as cyclooxygenases (COX), lipoxygenases (LOX) and cytochrome P450s (CYP), which produce soluble eicosinoids (e.g., thromboxanes, prostaglandins and other oxylipins) [7]. These oxylipins initiate a multitude of signaling pathways either as extracellular, high affinity ligands for GPCRs or as intracellular modulators of downstream effectors. In addition to signaling, the oxidation of ether-linked PUFA may promote iron-dependent cell death or ferroptosis in renal and ovarian cancer cells [130]. In parallel with PUFA derived from plasmalogens, the lysoplasmalogen backbone remaining after cPLA2-mediated hydrolysis has been implicated in immune cell functions such as phagocytosis [131] and “self” tolerance in the thymus [132]. Lysoplasmalogens can also be converted to major signaling lipids, namely alkyl-LPA and PAF. Even the alkyl chain at the *sn*-1 position of plasmalogens contributes to signaling, since liberation of this moiety by AGMO or a related enzyme generates fatty aldehydes that impact c-Jun N-terminal kinase (JNK) signaling and apoptosis [133]. Thus, the alkyl and acyl chains and the phospholipid backbone of plasmalogens ultimately serve as membrane storage precursors for signaling lipids released by lipase or monooxygenase activities, rather than direct components of signal transduction cascades. 

## 2. Conclusions

Despite their discovery almost one hundred years ago, the primary function of ether lipids remains in large part a mystery. It is clear that this class of lipids is ancient as they can be found in the most primitive of Archaea that dwell in some of the harshest environments on the planet. Their robust structure may well aid the ability of these organisms to resist environmental stress, modulate their membrane composition and adapt to inhospitable habitats. This cannot be their singular role, however, since they are synthesized in organisms that do not face extreme conditions from soil-dwelling bacteria to metazoan eukaryotic cells. In eukaryotes, ether lipids populate many different lipid classes, including fatty acids, glycerolipids, glycerophospholipids saccharolipids and GPI-linked lipids, performing unique functions in each. Perhaps not surprisingly, this taxonomic diversity coupled with representation in structurally unrelated lipid classes does not suggest a unified function for ether lipids, but rather points to a multifactorial utility.

Even though ether lipids were initially dismissed as odd constituents of lipid bilayers that alter the physico-chemical properties of cellular membranes, it is now clear that ether lipids dynamically participate in signal transduction pathways, among others. Ether lipids such as PAF and alkyl-LPA are indisputable ligands for specific GPCRs and potently elicit biological effects during complex physiological events such as inflammation and coagulation. More recently, metabolic intermediates of PAF such as HAG and HG have gained attention as signaling ether lipids that modulate PKC activity independent of their contribution to PAF biosynthesis. This raises an interesting question as to whether lipid intermediates that have dual functions are outliers or whether this represents a universal theme that has not yet been substantiated by more relevant examples of ether lipids impacting common signaling pathways. This question seems addressable, since the current momentum of ether lipid research appears to be increasing. In this context, the relatively recent discovery of the “ether lipid biosynthesis” (*elb*) genes in myxococci bacteria may be a landmark achievement, perhaps akin to the discovery of GNPAT and AGPS in the peroxisome. This area of investigation will likely open the door to more intriguing research in that domain, which may be applicable to other organisms and vice versa. 

Even within the signaling arena, there are many more open questions than answers regarding ether lipids. For example, the metabolic fate of ether lipid precursors such as alkylglycerol and their incorporation into cellular lipidomes has not been comprehensively defined. Alkylglycerol metabolism has only been examined in a few cell types to date, with platelets and smooth muscle cells metabolizing this lipid differently. Therefore, fundamental knowledge about existing lipid biosynthetic pathways in various cell types is lacking and may be cell type-specific. This knowledge gap is especially evident when relationships between the ether lipidome and seemingly unrelated lipid classes are revealed by manipulation of peroxisomal enzymes and systems approaches. Lipidomics is essential for identifying these relationships and for generating targeted hypotheses focused on causal mechanisms and potential metabolic overlap.

Another open signaling question is whether synthetic ether lipids can be effective therapeutics in human patients and provide protection against neoplasia or neurodegenerative diseases. Evidence from a multitude of studies demonstrates the efficacy of synthetic ether lipids in cell-based assays, xenografts and early stage clinical trials, but none have yet achieved Food and Drug Administration (FDA) approval.

Finally, high-resolution system approaches alongside hypothesis-driven strategies are poised to discover additional ether lipids and novel metabolic interrelationships between ester and ether lipidomes in diverse cell types. These approaches will likely clarify existing theories of ether lipids as dynamic membrane components, bioactive metabolites and signaling modulators, while simultaneously expanding the physiological relevance of these lipids beyond our current understanding.
